# The SEED Wellness Model: A Workplace Approach to Address Wellbeing Needs of Healthcare Staff During Crisis and Beyond

**DOI:** 10.3389/frhs.2022.844305

**Published:** 2022-04-01

**Authors:** Padmini Pai, Katarzyna Olcoń, Julaine Allan, Andrea Knezevic, Maria Mackay, Lynne Keevers, Mim Fox, Anne Marie Hadley

**Affiliations:** ^1^Illawarra Shoalhaven Local Health District, Wollongong, NSW, Australia; ^2^School of Health and Society, University of Wollongong, Wollongong, NSW, Australia; ^3^School of Nursing, University of Wollongong, Wollongong, NSW, Australia; ^4^NSW Ministry of Health, Sydney, NSW, Australia

**Keywords:** workplace wellness, healthcare providers, COVID-19, burnout—professional, mental health and wellbeing, occupational trauma exposure

## Abstract

Workplace wellness has gained new meaning and significance in the healthcare workforce in the face of the COVID-19 pandemic. Healthcare workers across the world have carried the burden of responding to the public health crisis by having to work under new pressures and constantly changing environments, take on additional shifts, risk their own health and lives, and cope with the ongoing psychological and emotional strain. The purpose of this paper is to articulate a workplace wellness model applied across hospitals in the Illawarra Shoalhaven Local Health District, a regional area in New South Wales, Australia. The description of the development, components, and lessons learned from the SEED Wellness Model illustrates one possible solution about how to provide better care for the staff thus not only preventing staff burnout and turnover, but also creating lasting organizational benefits. The detailed model description can assist in developing a larger and more rigorous evidence-base to improve staff wellness in healthcare settings, both within Australia and internationally.

## Introduction

Work takes up a significant amount of time in most adults' lives. The impact of work on physical and mental health has been extensively studied identifying risk factors for illness and injury (1, 2). As a result, many employers and governments have sought ways to ameliorate negative impacts of work by implementing regulations around responding to injuries and supporting return to the workforce (3, 4). More recently, ways of preventing work related harms have been investigated with a focus on wellness strategies such as health promotion campaigns (5).

The Global Wellness Institute (6) defines wellness as the active pursuit of activities, choices and lifestyles that lead to a state of holistic health. Although “wellness” and wellbeing” are often used interchangeably, we use the term “wellness” in this article as it relates to “an intention, action, and activities,” which is more appropriate for a workplace intervention, whereas wellbeing refers to a “perception of a state of being” (6). Workplace wellness from a health promotion approach typically focuses on individual risk factors and lifestyle change, such as increasing exercise. In this paper, workplace wellness is further conceptualized from a eudemonic perspective whereby people derive meaning and purpose from their work that includes (1) a perception that their role is important in the healthcare system, (2) experiencing trusting and personal relationships with co-workers; and (3) opportunities to contribute and grow within their role and the organization (7, 8). Workplace wellness is therefore not an individual experience or responsibility to address lifestyle risk factors, but a collective endeavor. Supporting this approach to workplace wellness requires systems and organizational actions as well as work-based interpersonal relationships that do not drain personal resources in the face of work demands.

Workplace wellness has gained new meaning and significance in the healthcare workforce as a result of the COVID-19 pandemic. Healthcare workers across the world have carried the burden of responding to the public health crisis by having to work under new pressures and constantly changing environments, take on additional shifts, risk their own health and lives, and cope with the ongoing psychological and emotional strain (9, 10). Studies on healthcare staff across the world demonstrate increased stress and anxiety (10, 11), emotional exhaustion (12), compassion fatigue and burnout (13, 14), a higher intention to leave the organization (15) and an overall reduction in the quality of care delivered due to COVID-19 (16). These studies unanimously call for workplace interventions to address the negative impact of COVID on healthcare staff and to prevent the onset of burnout and support staff retention (11, 12).

The purpose of this paper is to describe a workplace wellness model called SEED applied across healthcare settings in the Illawarra Shoalhaven Local Health District, a regional area in New South Wales, Australia. The Illawarra Shoalhaven Local Health District (ISLHD) extends across Yuin and Dharawal Country (the Indigenous traditional owners' names for the districts), about 250 km along the south-east coastline and services a population of more than 400,000 residents (17). ISLHD is one of the region's largest employers with a workforce of more than 7,300 across eight hospital sites and community health services (17). Following a discussion of the importance of wellness in healthcare settings, the article describes 1) the development and evolution of the SEED program, 2) “what is SEED?” - a summary of SEED principles and activities, 3) “SEED in action” - recent SEED outputs and plans for the future, 4) key challenges and lessons learned. Four of the authors have been involved in the design, implementation, and promotion of SEED, five are academics at a regional university currently researching the SEED model, and one author played both roles. The investment and potential bias of the authors involved in SEED was discussed openly in the process of writing this article and counterbalanced by a critical analysis of the SEED Reports and artifacts by the authors not directly involved in the program.

## The Importance of Wellness in Healthcare Workforce/Setting

Increasing workplace related illness and injury among healthcare staff are becoming a global concern for healthcare systems (1). Burnout has come to be a consequence of working in the healthcare system (18). Burnout in the healthcare setting has been linked to the experience of compassion fatigue and vicarious traumatisation through repeated exposure to patients and their health problems (19). These experiences within the health workforce cross disciplinary boundaries having been noted in medical staff (20), emergency nurses (21), mental health workers (22) and social workers (23, 24).

Constantly changing work environments and added pressures emerging from COVID-19 are contributing to greater levels of stress and feelings of burnout and apathy experienced by healthcare staff (25, 26). Specifically, COVID-19 has compounded job stressors associated with workload demands including, access to personal protective equipment, increased work hours, and workforce shortages (27). A multinational cross-sectional study describing the prevalence and predictors of burnout in healthcare staff during COVID-19 reported that 67% of respondents were at high risk of burnout (28). In contrast, prior to the pressures of COVID-19 the reported incidence of high range work-related burnout was ~40% (28, 29). These statistics stress that there is an increasing demand for healthcare organizations to address burnout among staff and develop preventative interventions that promote collaboration and support staff retention.

There is growing evidence to suggest that addressing staff wellness in healthcare can have lasting benefits for individual staff members, their patients, and the organizations they work within (30–32). The British National Health Service (NHS) is considered to have paved the way toward supporting and sustaining staff health and wellbeing, commencing with the implementation of a 5-year workplace wellness program (30). Though the NHS program was aimed at improving the health and wellbeing status of staff, findings also demonstrated organizational improvements (30). Additionally, in response to the rapid spread of COVID-19 in 2020, UK hospital trusts set up Supported Wellbeing Centres to mitigate the psychological impacts on healthcare staff (33). Wellbeing, work engagement, and job satisfaction were notably higher in those that accessed a wellbeing centre (33). Facilities were highly valued, but the service model was resource intensive with 134 wellbeing buddies supporting the centres in pairs (33). From this experience, it is evident that managerial support, financial and human resources, and advocacy are key to the success and sustainability of workplace wellness interventions (9, 33).

Although wellness is sometimes perceived as an individual responsibility that should be addressed outside the workplace (32), organizational interventions that aim to improve the wellbeing of employees have been found to increase productivity, decrease absenteeism, lead to greater job satisfaction, and increase commitment to the organization (30). Interventions designed to address wellbeing in the workplace include mindfulness training, social cohesion wellness programs, coping strategies, systems-wide approaches, and organizational culture of collaboration programs (34). In addition to preventing staff burnout and turnover, addressing workforce wellness can have lasting organizational benefits. Specific to healthcare organizations, early findings suggest focusing on staff wellness ultimately leads to better care and outcomes for patients (35). However, continuous evaluation of interventions is key to sustaining wellness in the workplace (34).

A shift to wellness promotion instead of a sole emphasis on responding to burnout requires a different focus from healthcare organizations. Workplace change is dependent on leadership, strategic, policy and operational support to embed and sustain new or altered practices (36). In Australia, the NSW Ministry of Health has stated it will invest in wellness through meaningful partnerships with patients, carers, and communities (37). As part of the partnership strategy the Ministry has implemented Elevating the Human Experience (ETHE) (38), a human-centered workplace culture program for those delivering care designed to impact those receiving it. The fundamental belief underpinning the ETHE program is that a positive workplace culture is a key enabler of good patient and carer experiences, and facilitates compassionate, and kind care. ETHE principles state “In taking care of those who provide care, we will excel at taking care of those who seek it” (38).

The ETHE program supports personalized staff interactions with each other, patients, and carers because this can significantly influence patients' and carers' experiences of care. While the NSW Ministry of Health has a state-wide focus, ETHE also aims to understand local contexts that staff work in to facilitate culture change. This includes place - the workplace environment, resources, community; processes - communication, feedback, and technology; and people – leadership, governance and staff experience (38). However, ways of tailoring wellness activities to local contexts have to be determined site by site. The Illawarra Shoalhaven LHD's SEED program has provided a demonstration of ways workplace wellness can be operationalised that could be replicated in other places.

## The Development and Evolution of Seed

Included in the ISLHD Strategic Directions is supporting, developing, and inspiring the workforce and enhancing the workplace culture (39). These strategic directions became of particular importance with the cumulative impact of natural disasters that affected the region in the last few years followed by the COVID-19 pandemic. The southern part of the district was heavily impacted by the 2019–2020 Australian bushfires. In New South Wales 5.5 million hectares burnt, with 2,500 homes destroyed and an estimated 800 million native animals killed (40, 41). The fires lasted for 74 days until being extinguished by heavy rains in February 2020. These rains in turn resulted in another disaster - unexpected floods.

Milton Ulladulla Hospital, a small hospital in the southern part of ISLHD, played a critical role in supporting the local community. As a large portion of the hospital staff reside in the same community and were personally affected by the bushfires and floods. The Chief Executive of ISLHD mobilized additional resources to respond to the psychological impact on the staff. The result was a locally designed and implemented wellness program the staff called SEED (Stability, Encompassing, Endurance and Direction) (42).

The SEED initiative was led by a Senior ISLHD staff member with experience in social work, change management, and leadership education in close collaboration with the Director of Nursing/Operations Manager and all staff of the Milton Ulladulla Hospital. The Program Lead initiated focus group discussions and individual consultations with hospital staff to learn about their needs and their vision for workplace wellness following the bushfires. Forty-one staff were consulted within 2 days, and 84 suggestions were themed into five workplace wellness initiatives. The co-design of program initiatives and the immediate follow-through resulted in the staff sense of empowerment, trust, and investment in the process (42). A final focus group was conducted with the goal of naming the initiative. The staff chose the word “SEED” to represent stability (staff need for stability moving forward after natural disasters), encompassing (wanting all their colleagues to be encompassed in the various initiatives), endurance (working together to endure the aftermath of the fires) and direction (having a sense of direction for self and for the organization). The initiative was intended to run for three consecutive months and to cover staff across all shifts (for details on the SEED activities and outcomes in the Milton Ulladulla Hospital read) (42).

As other hospitals in the district expressed an interest in SEED, the peer support initiative quickly grew to a workplace wellness model, with a longer-term focus on staff wellness and the development of a workplace culture of connection, compassion, and gratitude (43). As one of the members of the Executive Team expressed: “SEED may have had its genesis in fire, but what is evident is its relevance and transferability” (44). While acknowledging and adapting to the unique needs of each hospital, ward, and team, SEED has been introduced across the local health district to respond to the staff's wellness needs. For example, SEED has supported staff at another site as they transitioned to a new hospital building and during an unexpected loss of a beloved team member. Finally, SEED activities have offered support to staff during the stressful time preparing for and responding to the COVID-19 outbreak.

## What is Seed?

SEED is a workplace wellness model that strives for staff to experience more meaning, happiness, and connectedness at work. SEED was designed to produce and promote holistic wellness, a wellness of individual spirit, a wellness in community, a collective wellness of belonging, and a connection to land after the bushfire. The underpinning philosophy of SEED uses a strengths-based approach to ascertain the needs of staff and implement staff-led wellbeing initiatives that build resilience and aid recovery processes. In a strength-based approach the emphasis is on doing more of what works rather than trying to change deficits (45). The following quote from the Program Lead captures some of the key values and principles which are reflected in SEED:

Three important characteristics of my own leadership style have guided me throughout this time: mindfulness, compassion, and service. Practicing mindfulness and daily reflection has supported me in becoming more self-aware and focused. Compassion for me is not about being soft, but the ability to understand people at an emotional level, and truly caring about their wellbeing. Being committed to serving others during this time has helped me provide the space and support for my colleagues to thrive (43).

SEED is modeled around authentic participation (46) as well as self-determination, community involvement, empowerment, and inclusiveness. Although providing support for staff during the acute phase of bushfire crisis was initiated “top-down,” the model's essence, structure, and its name were co-designed with the hospital staff. The staff remained in control of the level to which they participated, and they were free to disengage at any time. The person-centered participatory approach has been crucial to support SEED's development as well as the localized and diversified implementation across the local health district. Learning through lived experience, critical reflection, and dialogue (46) are evident in the methods applied through all SEED activities and initiatives. Listening to self and to others is one of the foundational elements of SEED.

Some of the SEED initiatives that were implemented across different ISLHD sites include:

- Quiet Room - a space inside the hospital for staff to take a moment each day for a quiet reflection.
- Reflection Tree - hand-crafted wooden tree placed on the hospital wall signifying the growth of the hospital community following the bushfires. It has been adapted in other hospitals to a Kindness Tree, Gratitude Tree, Hope Tree where staff write on sticky notes one act of kindness they experienced at work or something they feel grateful for at work and hang it on the tree.
- Coffee Buddies - a planned coffee break with a nominated staff member as a form of collective care for each other. Buddies are randomly chosen from all staff and not organized by role or seniority.
- Wellness Warrior Training - a dedicated group of health care staff trained in strengths-based ways of creating a supportive work environment. For example, purposefully spending time listening to colleague's concerns and experiences without jumping to solutions.
- 24/7 Wellness - facilitating 24 weekly wellness sessions in the workplace to promote staff wellbeing [for details, see (42)].
- (S)Crap book- a unique communal journal designed by the staff and placed in the tearoom for front line staff to take a moment of reflection. The book aims to facilitate externalizing their unique experience and stressful stories occurring during the pandemic.

New ideas or adaptations of the existing ideas are encouraged at each site to respond to the unique needs and circumstances of the staff.

Finally, SEED is also inclusive of the leadership teams by 1) inviting leaders to all SEED activities and 2) holding leadership meetings and workshops to create space for leaders to reflect on their leadership style, interactions with staff, and vision around staff wellness for the future. Leaders are welcome to join any wellness activities and are free to disengage at any time. Importantly, SEED has been intentional in not replicating the hierarchical relationships while engaging in SEED activities; as explained by the Program Lead: “wellness is a space where we sit as equals” (43). The SEED leadership workshops in turn challenged traditional ways of working by querying how leaders think about challenges and solutions, what leaders do to make a difference, and where leaders need to focus efforts. Ultimately, SEED promotes a collective model of leadership through intra- and inter-hospital connection and collaboration.

## Seed in Action

Since the conception of SEED in January 2020, over 600 staff and 40 leaders have been involved in SEED activities and events. SEED sessions have been conducted to create awareness around the concept and importance of workplace wellbeing. Other sessions have included mindfulness, mentoring, facilitation, education sessions and information sessions. At a local level, the community has become involved in SEED initiatives. As the COVID-19 pandemic forced intensive care nurses into isolation, community members cooked and delivered meals to them after SEED participants put out a call to friends and neighbors. The most recent SEED events were the World Kindness Day celebrations in hospital wards and the coming together of 35 staff and few community members to be part of a Visual Story Telling event. The collective story captured the journey of SEED, its personal impact as shared by participants and identified where this group visualizes SEED moving forward including expanding to new sites.

Ways of sustaining and growing SEED has been a focus of 2021. SEED is now part of change preparedness activities as staff are introduced to the collection and use of Patient Reported Measures (PRM) in the inpatient settings. ISLHD is the first site in NSW to launch PRMs *via* an IT platform called HOPE (Health Outcomes Patient Experience). The SEED model approaches PRMs implementation by firstly focusing on staff wellness, which in turn may help them engage more effectively with their patients. Recognizing that constant workplace change causes stress and uncertainty for staff, SEED will support the pilot wards to enhance staff wellness as a prerequisite of effectively using HOPE. This SEED initiative is now part of a PhD study in collaboration with University of Wollongong (UOW) to formally evaluate the role of staff wellness in HOPE implementation.

More broadly, SEED has garnered interest at a state government level with several SEED wellness sessions delivered to staff at the NSW Ministry of Health. The concept of SEED has been presented at three major conferences and several celebration days including World Social Work Day and The Compassion Revolution Conference. Other ways of sharing the SEED model have included creating audio visual materials that portray staff experiences and articulate ways SEED has been practiced. For example, three podcast episodes have been completed and launched on The Social Work Stories Podcast in November 2021 (47), a 30-min documentary film and a further 3-min excerpts are available to share the SEED story (48).

## Key Challenges and Lessons Learned

SEED design and implementation has not been without challenges and there are several lessons that were learnt in the process. SEED began immediately after a bushfire disaster and there was a high level of suspicion and reluctance expressed in the start-up phase from the hospital staff and management. Strong consultation and facilitation skills from the founding SEED team were thus essential to work through those initial responses and encourage people to become involved. Still, the wellness activities were voluntary and not all staff participated, leaving some staff expressing disconnection and exclusion. The lesson here has been to work closely with those staff experiencing disconnection, and to ensure that all staff are given explicit permission and encouragement from the hospital leadership to look after their wellness during work time. More importantly, organizational measures need to be put in place that alleviate a sense of guilt that staff may feel when they prioritize their own wellness in work hours. To mitigate some of these issues, SEED wellness activities were scheduled at a time where there was one-hour overlap between changing shifts, meaning theoretically no floor staff should have been burdened by scheduled wellness activities. Still, the timing and the types of wellness sessions were not able to accommodate everyone in what was a diverse staff group.

A further challenge encountered was that the staff-led and organic nature of SEED did not fit neatly into the way healthcare settings usually operate. The participatory methodology of SEED required flexibility, patience, and was process-oriented, creating potential clashes with the tick-box, outcome-oriented healthcare culture. Questions therefore present in relation to the potential expansion of SEED beyond the original hospital – how do you scale a staff-led program up without losing its organic and situated character? Although ideas of wellness activities have been described in this article, all SEED activities are formulated by the staff themselves, making them unique to their setting. Accordingly, to stay true to its core principles, SEED cannot be manualised and summarized into a step-by-step approach. Although there are core SEED practices that can be followed, each team/ward/hospital must “plant its own SEED” by designing its localized SEED wellness activities.

A final challenge SEED encountered was related to resources. Although SEED is a relatively low-cost workplace wellness intervention, it requires certain human, physical and fiscal resources including a facilitator or a team of facilitators, depending on the size of the workplace, who can guide the start-up phase at each site. The length of this phase varies depending on staff availability and engagement. SEED follows the “train the trainer approach” as it identifies and trains staff at each site who can take the lead in implementing and maintaining SEED activities. In addition to covering the cost of the facilitators and staff attendance at the consultation and training sessions, workplaces need to be willing to dedicate a certain number of hours weekly to the identified staff members to organize and implement the SEED activities. If the site is understaffed, as has been the case in many hospitals during the COVID-19 pandemic, the likelihood of prioritizing staff wellness decreases. SEED has encountered resourcing challenges at various stages and sites have had to look for alternative ways of ensuring the continuity of the wellness initiatives, such as community and local university support. The spread and sustainability of wellness initiatives thus depends in large part on the ongoing access to resources.

## Conclusion

The description of the development, components, and the process of the SEED Wellness Model illustrates one possible solution about ways to provide better care for healthcare staff thus not only preventing burnout and turnover, but also creating lasting organizational benefits. While starting prior to the NSW Ministry of Health Elevating the Human Experience strategy, SEED has operationalized the strategy by creating a localized (place), staff designed, and leadership supported (people) wellness plan (process) that is human-centered. The organic development of the SEED wellness model as a collective crisis response; and its subsequent staff engagement and repurposing for new sites demonstrates an appetite for such initiatives among healthcare staff.

Although there is a growing number of workplace interventions to promote wellness such as the 5 Ways to Wellbeing (49), the PERMAH Wellbeing Lab (50), and the 5-year workplace wellness intervention in the NHS (30), we assert there are several features that make SEED distinctive. For example, SEED does not follow a “one size fits all” approach, as the intervention is co-designed at each site with the staff to meet their specific needs and interests. The model takes a team-based approach to wellness that promotes a supportive workplace atmosphere to foster collective resilience, in contrast to wellness initiatives which tend to focus on individual risk factors and lifestyle changes. SEED promotes a workplace culture where staff are given the permission and are encouraged to look after their wellbeing during work time. Finally, SEED has a non-hierarchical and inclusive nature which provides a space where staff members at all levels are being treated as equals.

The detailed model description provided in this article can assist in developing a larger and more rigorous evidence-base to improve staff wellness in healthcare settings, both within Australia and internationally. An empirical study which examines SEED practices and outcomes in response to pandemic-related stress is already underway. This study is using qualitative methods to explore SEED participant's experiences of the program. But there remains a need for more research. Specifically, research investigating the barriers and enablers to SEED, identifying what are core components in all sites and evaluating the impact of SEED using staff and organizational wellness measures, including calculating cost effectiveness, will refine the model and facilitate transferability. A stepped wedge cluster randomized trial at eight SEED implementation sites is planned for 2023.

Created to respond to the needs of hospital staff in the aftermath of bushfires, the SEED Wellness Model has now been applied across four hospitals in the Illawarra Shoalhaven Local Health District and is gaining state-wide attention in Australia. The practices that have underpinned the growth of SEED have successfully been adapted to a variety of contexts. These practices include hospital leadership being open to listen in a way that identifies the collective needs of staff, creating wellness interventions that respond to these identified needs, and enabling staff to become “wellness warriors” who are open to companioning their colleagues by listening to their experiences of hurt and trauma. The practices of SEED have resulted in creating positive workplace cultures where staff have described a sense of human flourishing. Staff designed wellness programs can offer healthcare organizations an alternative to ameliorating burnout amongst healthcare workers, particularly during a crisis such as a natural disaster and pandemic. Just as we need to prevent natural disasters such as the Australian Black Summer, we need to strive to prevent healthcare staff burnout. As SEED has demonstrated, promoting and maintaining wellness initiatives is perhaps the best way to go about it.

## Author Contributions

PP, KO, and JA developed the paper scope and aim, wrote initial drafts, and finalized the submission. AK, MM, MF, LK, and AH each drafted a section of the manuscript, reviewed drafts, and approved the final version. All authors agree to be accountable for the content of the work.

## Funding

This project was jointly funded by the University of Wollongong 2020 Community Engagement Grant and the National Health and Medical Research Council (NHMRC) through the Medical Research Future Fund (MRFF) 2020 COVID-Mental Health Research Grant MRF2005659.

## Conflict of Interest

The authors declare that the research was conducted in the absence of any commercial or financial relationships that could be construed as a potential conflict of interest.

## Publisher's Note

All claims expressed in this article are solely those of the authors and do not necessarily represent those of their affiliated organizations, or those of the publisher, the editors and the reviewers. Any product that may be evaluated in this article, or claim that may be made by its manufacturer, is not guaranteed or endorsed by the publisher.
